# *In Vitro* Effects of Low-energy Ultrasound Treatment on Healthy CD3/CD8+ Lymphocytes, Red blood cells, Acute Myeloid leukemia cells, and Jurkat cell line

**DOI:** 10.7150/jca.83050

**Published:** 2023-04-24

**Authors:** Francesco De Leonardis, Simona Nicole Barile, Claudia Cianci, Isabella Pisano, Giuseppe Merla, Giovanni Pappalettera, Caterina Casavola, Carmine Pappalettere

**Affiliations:** 1 Dept. Bioscience Biotechnology and Environment, University of Bari” A. Moro”, Bari, 70125, Italy.; 2 Department of Molecular Medicine and Medical Biotechnology, University of Naples Federico II, Naples, 8013, Italy.; 3 Dept. of Mechanics, Mathematics, and Management, Polytechnic University of Bari, Bari, 70125, Italy.

**Keywords:** Lymphocytes, Red blood cells, CD3/CD8 lymphocytes proliferation/activation and cytotoxic functions

## Abstract

The study of the biological effects of low-energy ultrasound and its applications is a rapidly expanding research area. Low-energy ultrasound could be used as anti-tumoral therapy with or without the pharmacological combination even if the second situation has been scarcely investigated up to now. Very little information is available about the ultrasound effects on healthy red blood cells, CD3, and mainly CD8 subset lymphocytes which is the main subset cell having cytotoxic function towards cancer cells. In this study, we investigated *in vitro* the bioeffects of low energy ultrasound on red blood cells and PBMCs isolated from healthy donors as well as on two myeloid leukemia cell lines (OCI- AML-3 MOLM-13) and lymphoblastic Jurkat cell line. Using low-energy ultrasound (US), a study was conducted to determine how it affects CD3/CD8 lymphocytes and leukemia cells, as well as its potential role in treating blood cancers, by analyzing changes in mitochondrial membrane potential, phosphatidylserine asymmetry, morphological changes for myeloid AML cell lines, proliferation and cytotoxic activation of healthy lymphocytes, and apoptosis for RBCs after US exposure. Overall, we demonstrated that CD3/CD8 lymphocytes proliferation/activation and cytotoxic functions are fully preserved after ultrasound treatments, whereas leukemia cell lines undergo apoptosis and stop proliferating suggesting a potential method of treating blood cancer.

## Introduction

The Ultrasound (US) has a great potential for therapeutic applications, specifically for induction of apoptosis in cancer cells, and drug delivery 1-4. In particular, the interest for the adoption of low-intensity treatment is related to its potentiality of selectivity connected with different resonance frequencies of the healthy and malignant cells. In a general way, the interaction of ultrasound with a cell culture can produce three distinct effects: microbubble formation, hyperthermia, and resonant excitation 5, 6. While cavitation and thermal effects cannot provide selectivity, resonant excitation can act differently on healthy and cancer cells due to their distinct morpho-physiological effects. Working at low intensity regime can inhibit cavitation and thermal effects and promote resonant ones. Frequency resonance can change in healthy and cancer cells due to modification in mechanical properties. Change in stiffness in cancer cells, in fact, has been demonstrated with a reduction of the rigidity that can amount up to 70% 7. Based on this consideration Fraldi et al. 8 have developed a specialized viscoelastic model of the healthy and malignant cells demonstrating a significant shift in the resonance frequency on many different cells. In 9 the frequency resonance hypothesis is illustrated even in terms of possible effects on subcellular structures and enzymatic activity. However, a complete understanding of the effects of ultrasound on cells has not been achieved yet. In this general framework, Pappalettere et al. 10,6 tested the application of low-intensity US on U937 human myelomonocytic cells comparing the effects, in terms of rate of survival, when operating at fixed frequency rather than sweeping. Selectivity of US was also investigated comparing the viability post-treatment of MCFT7 (human breast adenocarcinoma) and MCF10A (healthy breast cells) also suggesting a correlation between the effects of the US treatment and the variation in mechanical properties (e.g., stiffness) of the cancerous cells 11. It should be underlined that, in the cited works, US was used as a stand-alone approach without synergic adoption of chemotherapy treatment. Apoptosis induced by low intensity US can be associated with acoustic cavitation and mechanical resonance effects induced by ultrasound 12-14. Clinical application of low-intensity ultrasound has been hampered due to an imprecise understanding of its apoptotic and genotoxic activity in both healthy and malignant cells 15. Several studies have reported the effects of low-energy treatment on myeloid leukemia and lymphoblast cells 15-17. Ultrasound induces DNA damage and apoptosis through the formation of reactive oxygen species such as hydrogen peroxide and hydroxyl radicals 18, 19. It's unclear if ultrasound causes epigenetic modification or gene expression alteration, abnormal proliferation/activation mechanisms, differentiation, or defective cytokines- granzymes production on healthy lymphocytes.

Activation of T cells, a major fraction of peripheral blood lymphocytes, is essential for the immune response against cancer cells. CD69 and CD137 are important for the surviving, expansions, and cytotoxic activation, as well as proliferation and differentiation of healthy lymphocytes. CD69 is a surface marker receptor that appeared on activated lymphocytes after stimulation. CD69 regulates the differentiation of regulatory T (Treg) cells and secretion of some cytokines such as IFN-γ, IL-17, and IL-22. The appearance of CD69 on the plasma membrane of activated cells is faster, underlying its widespread use as an early marker of lymphocyte activation 20, 21. CD69 also interacts with LAT-1 (amino acid transporter, tryptophan transporter) 22 suggesting its functional role in switching metabolism during activation.

CD137 (4-1BB) is a surface glycoprotein that belongs to the tumor necrosis factor receptor family (TNFRSF9) 23. CD137 becomes expressed on primed T and natural killer (NK) cells. Interaction of CD137 by CD137L on activated CD8 T cells protects cytotoxic T lymphocytes from apoptosis, enhances effector functionalities, and favors persistence and memory differentiation 23. CD137 becomes expressed on primed T and Natural Killer cells and recent studies have demonstrated that CD137 ligation causes strong costimulatory signals in T cells and thus rising IL-2, interferon-gamma (INF-g), and other cytotoxic cytokines. Furthermore, CD137 has protective roles in preventing apoptosis in T cells as well as cell memory and effector differentiation changes the mitochondrial metabolism to increase T-cell respiratory capacities. Finally, CD137 expression also induces DNA demethylation and reprogramming gene expression 23.

Granzymes are serine proteases released by cytoplasmic granules within cytotoxic T cells and natural killer (NK) cells. They induce programmed cell death (apoptosis) in the target cell, thus eliminating cells that have become cancerous or are infected with viruses or bacteria 24. Here we demonstrated that in our condition the US treatment can kill leukemia cell lines without affecting all functions of healthy lymphocytes. In this work, we have evaluated the viability, proliferation/expansion as well as cytotoxicity and activation of healthy CD8+ cells, after ultrasound treatment. Using Anti-CD3/CD28 magnetic beads as a superantigen (APC-like cells)/mitogen activation complex 25 we have evaluated the functionality of US treated lymphocytes, as the expression of CD69, CD137, and Granzyme B production. Finally, we analyzed mitochondrial membrane potential, expression of anti-apoptotic Mcl-1, ROS production, and GSH content after US treatments in healthy lymphocytes and leukemia cells.

## Materials and Methods

### Ethics statement

The samples were collected as part of NETWORK TELETHON DI BIOBANCHE GENETICHE [Genomic and Genetic Disorders Biobank] the use of volunteers' blood samples was approved by the institutional review board at IRCCS Casa Sollievo della Sofferenza, San Giovanni Rotondo, ethics committee cod. GTB.12001(GGDB). The study was conducted in accordance with the Declaration of Helsinki and approved by the Institutional Ethics Committee of NETWORK TELETHON DI BIOBANCHE GENETICHE (code GTB.12001 (GGDB).

### Informed Consent Statement

Written informed consent was obtained from all volunteers.

### Cell culture

Human myeloid leukemia cell lines (OCI AML-3, MOLM-13) were obtained from (Leibniz Institute DSMZ- German Collection of Microorganisms and Cell Cultures GmbH). For the lymphoid leukemia counterpart, we used Jurkat ATCC-TIB-152 clone E6-1. All cells were grown in RPMI-1640 (Gibco, Euroclone, ITA) supplemented with 10% FBS (Euroclone, ITA), 1% L-Glutamine (Gibco), and 1% Penicillin-Streptomycin (Gibco) except where indicated. RBC (Red Blood cells) and PBMC (Peripheral Blood Mononuclear cells) from heparinized venous blood were obtained from healthy volunteers. PBMC were separated from Peripheral blood by PBMC 24+ Spin Medium gradient (PluriSelect Life Science, Germany), then cultured in RPMI-1640 supplemented with 20% FBS for one day followed by fresh RPMI-1640 supplemented with 10% FBS. Volunteers were taken from healthy 25 to 60 years old and both sexes.

### Proliferation assay

To verify the effectiveness of US treatment in killing cancer cells, leukemia cell lines, UT and after 2T treatment, were grown for up 6 days. Untreated and US-treated leukemia cells (80000 cells) were grown in RPMI- 1640 in 96 well plates. To test the proliferation (*In vitro* expansion) of healthy PBMC we used Dynabeads since resting lymphocytes can proliferate only after stimulation, therefore lymphocytes (UT and 2T) from 3 healthy donors were grown in the presence of CD3/CD28 Dynabeads (Cat: 11161D Thermofisher, Life Technologies Italia) 25 for up 6 days as indicated by manufacturer.

### Proliferation rate of PBMC

UT and 2T PBMC were growth up to 9 days in the presence and absence of Dynabeads, then proliferation rate was calculated as ratio of cell at 9 days / cell at 7 days of cells grown in the presence of Dynabeads as indicated by manufacturer.

### Ultrasound apparatus

The system used to generate ultrasound is the KTAC-400 Sonoporation System (Nepa Gene, Japan). The KTAC-4000. The sonoporation transducer used in the experiments is the 20 mm diameter probe KP-S20 (Sonidel, USA).

### Experimental setups for ultrasound treatment of cultured cells

On the day of the experiment, just before the ultrasound treatment, cells were harvested and collected through centrifugation at 1500 rpm for 5 minutes at room temperature. Pellets were resuspended with a fresh complete medium at the density of 1 x 106 cells/ml as indicated by Feril et al. 26. The US transducer was placed in contact with the plate bottom after applying the coupling gel (Pharmaceutical Innovations Inc., NJ, USA) between the probe surface and the well bottom. The cells were treated with an intensity of 1 MHz, 50% duty cycle, for 20 s (T) or 40 s (2T), with a burst rate of 10 Hz, voltage 40 Volts. The relative I_STPA_ was from 38 to 51 mW/cm2.

### Flow cytometry Analysis

Flow cytometry data were obtained using Invitrogen Attune NxT 488 blue laser and BD FACS Melody cell sorter flow cytometers. All density plots are representative of 3 independent experiments. 10000 to 20000 events were acquired for all experiments except were differently indicated. Data analysis was performed with FCS Express Software V7, and FlowJo 10.4. Lymphocytes, leukemia cell lines, and RBCs were gated based on FSC-A vs SSC-A or FSC-H vs SSC-H parameters. Lineage markers used were anti-human CD3 Antibody PerCP-Vio® 700 (Miltenyi Biotec, Biotechnology Company Italia) or CD8a Monoclonal Antibody (SK1), PerCP-eFluor 710, eBioscience (Thermofisher, Life Technologies Italia) as indicated by the manufacturer. Lymphocytes were also stained with CD8+ activation marker CD69 Monoclonal Antibody (FN50), PE, eBioscience, CD137 (4-1BB) Monoclonal Antibody (4B4 (4B4-1)), PE, eBioscience™ and its isotype control Mouse IgG1 kappa Isotype Control (P3.6.2.8.1), PE, eBioscience. For intracellular staining lymphocytes were preincubated with the protein transport inhibitor cocktail (500X) from Thermofisher and used the following antibodies: Granzyme B Monoclonal Antibody (N4TL33), PE, eBioscience™ with isotype control Mouse IgG2a kappa Isotype Control (eBM2a), PE, eBioscience, and eBioscience™ and Intracellular Fixation & Permeabilization Buffer Set, as indicated by the manufacturer. RBCs were stained with CD235a (Glycophorin A) Antibody, anti-human, PerCP-Vio® 700, REAfinity™ (Miltenyi Biotec, Biotechnology Company Italia) as indicated by the manufacturer. Live lymphocytes were gated on basis of their FSC-H and SSC- H as indicated in 27 and by Annexin-V/PI analysis. Monobromobimane cod. CAY-17097-25 was from Caymanchem. Dichlorodihydrofluorescein diacetate (DCFH-DA) cod. D6883 was from Sigma Aldrich. Calcein-AM cod.65-0853-39 was from Thermofisher.

### Morphological studies

The cell viability was assessed by trypan blue exclusion test immediately after ultrasonic treatment and after 24 hours. The cell suspension was analyzed immediately and after 24 hours of treatment by light microscopy (Nikon Eclipse TS-100). Images were taken by CCD Camera mounted on light microscopy and using NIS-elements Imaging Software (Nikon).

### Calcein*-*AM viability assay

1 x 106 cells/mL of whole Blood cells, PBMC, and leukemia cells were treated with one (T) or two (2T) ultrasound (US) pulses, followed by 24 hours of incubation at 37 °C, CO_2_ 5%. 1,5 x 106 RBCs/mL collected and incubated with 5 μM of Calcein-AM for 135 min in RPMI 37°C following a Bratosin et al. modified protocol 28 and then stained with CD235a antibody or Annexin V. Leukemia cell lines and PBMC, briefly, the cells were collected after T and 2T treatments and resuspended in HBSS +/+ (Thermo Fisher, Life Technologies) at 106 cells/ml and incubated with 50 nM Calcein-AM for 20 min 37 °C, CO_2_ 5% in the incubator. Finally, lymphocytes were collected, washed with HBSS +/+, and resuspended with 20 ug/ml HBSS+/+ Propidium Iodide solution for 15 min RT. RBC were washed in PBS + 5% BSA then resuspended in same buffer and analyzed.

### Annexin V apoptosis assay

Cell lines, PBMC, and myeloid/lymphoblastic cell lines were analyzed 30 min and 24 hours after Ultrasound treatment (US), by Flow cytometry Annexin V Alexa Fluor 488 and Propidium Iodide (PI) assay using the Tali® Apoptosis Kit - Annexin V Alexa Fluor® 488 (Molecular Probes, Life Technologies) as recommended by the manufacturer. For Eryptosis assay, RBCs untreated and treated were resuspended in 200 μl of 1X Annexin Buffer plus 10 μl Annexin-V Alexa Fluor, then spun down at 800 x g 10 min washed with PBS + 0.1 % BSA and analyzed by Flow cytometry.

### GSH content assay

To assess GSH content in untreated or US-treated lymphocytes, the cells were incubated with Monobromobimane (MBB) as indicated in 29. Briefly, the 2 x 106 cells were incubated for 10 min 37 °C with 50 μM of MBB, then washed with PBS and analyzed for GSH content using FACS Melody a 405 laser and using a BV421 filter. For the negative control, lymphocytes were pre-incubated with NEM 100 μM in PBS, for 20 min 37 °C, then washed with PBS and added 50 μM MBB for 10 min 37 °C; Finally, the cells were washed in PBS and analyzed using FACS Melody.

### Mitochondrial membrane potential and Mitomass

The mitochondrial membrane potential of leukemia cell lines, PBMC, was estimated after 24 hours of treatment by Flow cytometry using Mitotracker red (Mitochondrial Potential) versus Mitotracker Green (Mitomass) assay (Thermofisher Scientific), according to a published protocol in Monteiro et al. 30. Briefly, the cells were resuspended at 5 x 105 cells/ml of PBS pH7.2 supplemented with 2% FCS then incubated for 20 min with 25 nM Mitotracker Red and 100 nM Mitotracker Green mix staining solution at 37°C or preincubated with 100 nM of ionophore Valinomycin 15 min before staining, as a positive control, were indicated. For flow cytometry analysis 15000 events were acquired.

### Mcl-1 expression

To evaluate Mcl-1 expression after 30 min and 48 hours, after US treatment, 106 cells were collected after US treatment and solubilized in sample buffer 2X and SDS-PAGE, followed by a Western Blot. A 1:1000 dilution of monoclonal antibody Mcl-1 (D35A5) cod.5453 (Cell Signaling) was used, as indicated by the manufacturer. Rabbit secondary antibody (peroxidase-conjugated) was used. As housekeeping normalization protein, homemade antibody raised in rabbit against AGC (SLC25A12 aspartate/glutamate carrier) antibody which recognize both isoforms AGC1 and AGC2, was used as indicated in 31, 32. Densitometric analyses were accomplished by using the Image Lab™ Touch software (Bio-Rad Laboratories, CA USA). All blots were cropped between 75 KDa (for housekeeping AGC protein) and 45-30 KDa for MCL-1 isoforms.

### Reactive oxygen species (ROS) determination

Lymphocytes were grown on 12 multiwells plates and at 106 cells/ml in RPMI-1640 supplemented with 10% FSC. After 30 min or 24 hours post-treatment, the cells were incubated with 5 μM DCFH-DA, for 30 min 37 °C, 5% CO_2_. After washing with PBS, the pellets were analyzed to flow cytometer. ROS contents were measured on live non apoptotic cells. 20000 events were acquired.

### *In vitro* Stimulation and Expansion of T Lymphocytes with anti-CD3/CD28 DynaBeads

For *in vitro* activation markers analysis, we used anti-CD3/CD28 DynaBeads + 40 U/ml recombinant Il-2, and recombinant Il-7 at 15 U/ml as described by the manufacturer protocol 25. Responder cells of all experimental conditions were counted at indicated days after stimulation and US treatments. For proliferation assay, the growth of untreated and US-treated lymphocytes was followed by incubation with DynaBeads, for up to 9 days. The cells were monitored and counted by trypan blue exclusion, before and after 9 days of incubation, and the images were acquired at 0, 7, and 9 days after US treatments. The proliferation rate was calculated as follows: stimulated (with Dyanbeads)/unstimulated (without Dyanbeads) ratio expressed as fold of proliferation (proliferation at 9th day/proliferation at 7th day) for untreated and treated cells.

### Early activation marker CD69 and CD137a cytotoxic marker expression and Granzyme B production

Lymphocytes were grown at 1 x 106 cells/ml in 12-well plate with RPMI-1640 supplemented with 10% FSC and treated with (2T) 20 s, then seeded onto a 24-well plate and followed by incubation of 24 hours as described above. The cells were then incubated with anti-CD3/CD28 DynaBeads 25, 40 U/ml recombinant IL-2 and, 15 U/ml of recombinant IL-7. Finally, after removing magnetic beads, cells were stained with anti-CD69-PE, and CD3 (FITC) or with anti-CD137a (PE), and CD8 PerCP-eFluor 710 and relative Isotype control as described by the manufacturer and analyzed by flow cytometry. Lymphocytes have been also grown-up for 48 hours with Dynabeads and interleukins, and for the last 4 hours with a protein transport Inhibitor cocktail then analyzed by intracellular staining for Granzyme B and specific isotype control.

### Jurkat cell cycle analysis

Jurkat cells, after exposure to US, were collected with an ice-cold PBS, fixed in cold 70% EtOH and incubated ON -20°C. Then, the cellular pellet was washed twice in PBS and treated with 50 µL of RNAse (100 µg/mL, Sigma). 500 µL of PI (50 µg/mL, Sigma) and 0,001% NP-40 were added. 15000 events were collected and analyzed.

### Statistical analysis

Unpaired *t*-test with Welch's correction and non-parametric Mann-Whitney two-tailed tests were used to compare group means except, where indicated, in Figure. 4. Data were plotted and analyzed by using GraphPad Prism software version 8.01. All experiments contain at least 3 biological repeats. The difference between the mean values was analyzed by paired Student's *t-*test if not otherwise indicated. P values less than 0.05 were considered statistically significant in this study.

## Results

### Susceptibility of healthy PBMC, RBCs, and AML cell lines to ultrasonic treatment

**Figure 1:**
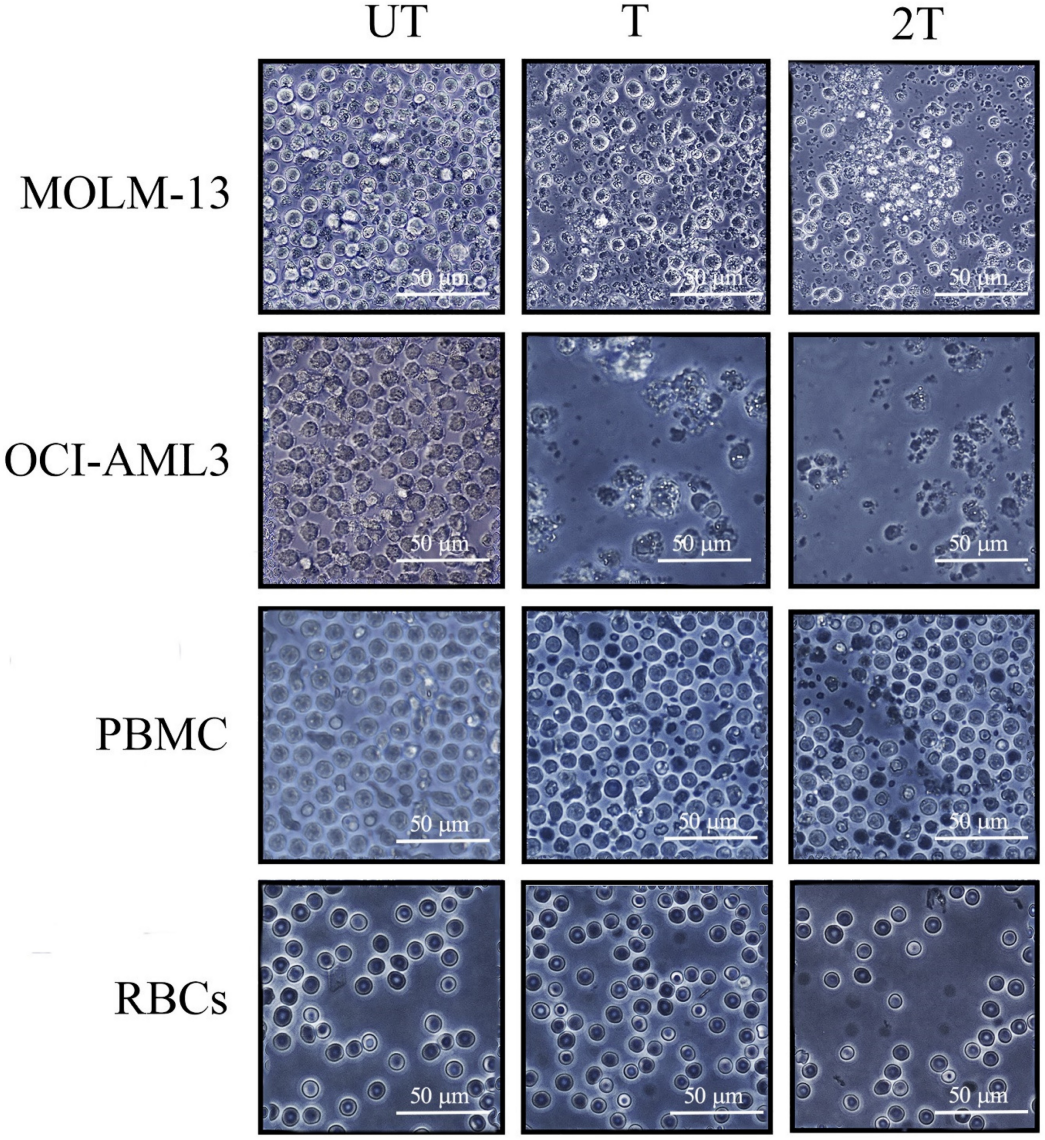
** Light microscopy images of leukemia myeloid cells (MOLM-13, OCI-AML-3), RBCs and lymphocytes of healthy donors 24 hours after US treatment.** 106 cells, UT untreated, T 20 s exposure, 2T 2 x 20-s exposure at 1 MHz, duty cycle 50% burst rate 10 Hz. The bar represents 50 μm of resolution at 40X of magnification. Representative images of three independent experiments

To test the effects of US on the viability of PBMC, myeloid and lymphoblastic leukemia cell lines, we set up the different conditions of US exposure. We used best condition of 1MHz and different time exposure (T=20 s of treatment and 2T= two exposures of 20 s each) of intensity to test the best condition to assay the mortality on leukemic cell lines as well as on healthy PBMCs and RBCs. Best mortality conditions for AML cell lines were obtained at 1 MHz, 50 % of duty cycle, and 10Hz burst rate using 2 exposures of 20 s (2T) as shown in Figure 1. Under 2T sonication treatments, all myeloid leukemia cells undergo apoptosis/necrosis events.

OCI-AML-3 cell line was more sensitive to all treatments and showed a necrotic effect to a very high extent compared to the untreated (UT) controls and any intact cells were visible. Furthermore, MOLM-13 cell line showed less necrotic effect after one treatment (T) but are still visible morphological variations (e.g., cell shrinkage, membrane blebbing, etc.). It's interesting to note that MOLM-13 cell line have more resistance to ultrasound, probably they could have a similar response to Molt-4 (another leukemia myeloid cell line) 33, or have different expression of P53 gene 34,35. This result is consistent with the “Oncotripsy” theory developed by Ortiz et al. 36.

On the contrary, very few visible apoptosis events, 24 hours after US treatments, were appreciable for healthy PBMC and RBCs, probably early apoptosis is reversible on lymphocytes 37. It's interesting to note that lymphocytes remain alive after US treatments without any anti- apoptotic cytokines such as IL-2 and IL-7. The results demonstrate that low-intensity US *in vitro* was able to induce selectively apoptosis/necrosis in myeloid leukemia cells, which are more subject to apoptotic effects of US, but not in healthy controls. Hence, AML cell lines are more susceptible to necrosis than healthy PBMC.

### Susceptibility of Jurkat cell line to ultrasonic treatments

**Figure 2:**
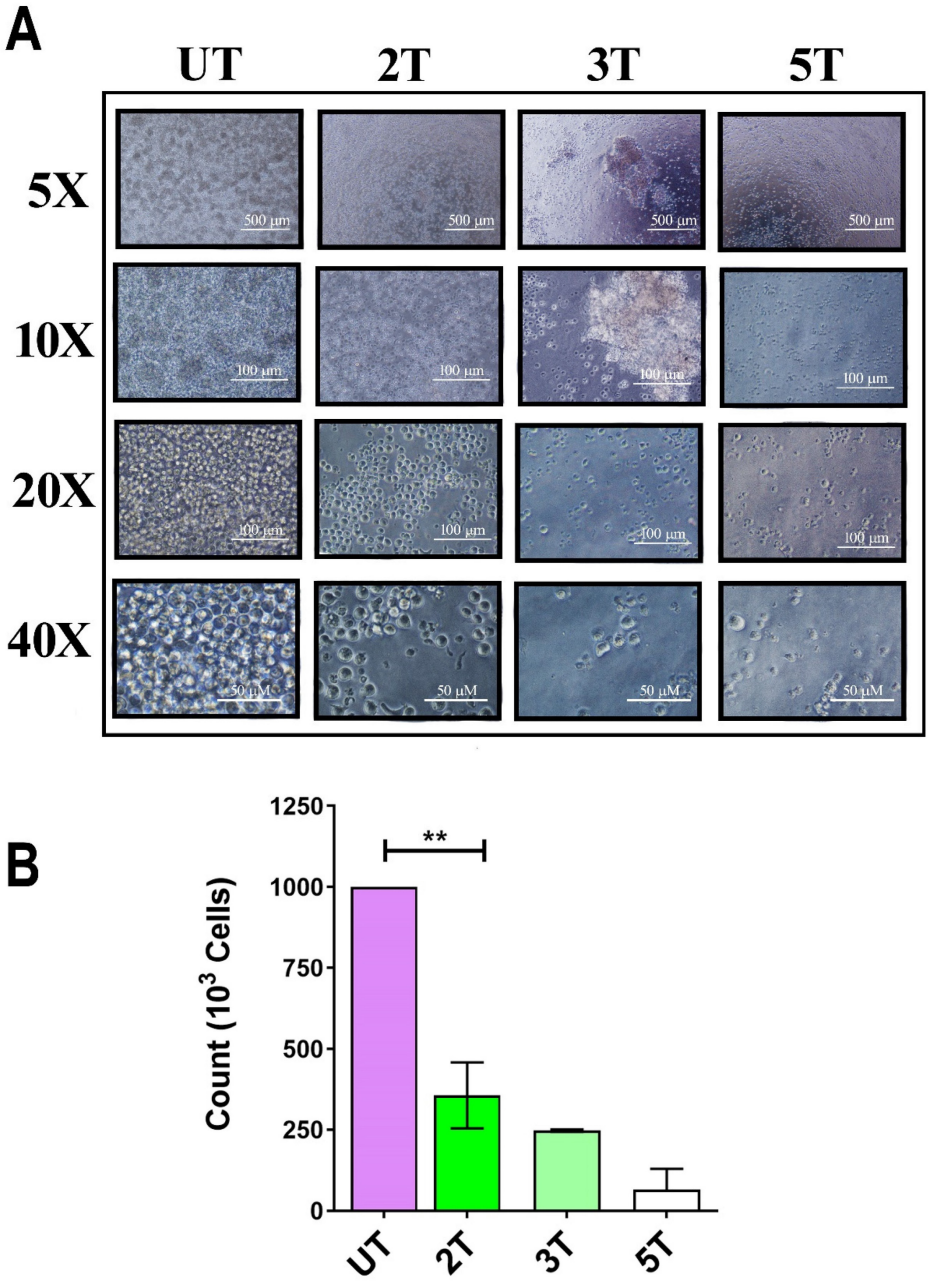
** Effect of ultrasound on Jurkat cells. (A*)*** 10^6^ Jurkat cells/ml were treated with ultrasound and live cells (trypan blue exclusion) were counted immediately after US treatment. 2T, 3T and 5T are 20, 60 and 100 s of US treatment, respectively. **(B)** histogram of live Jurkat after US treatments. The bars represent 500 μm (5X magnification), 100 μm (10X and 20X magnification) and 50 μm (40X magnification) of resolution.

**Figures 3:**
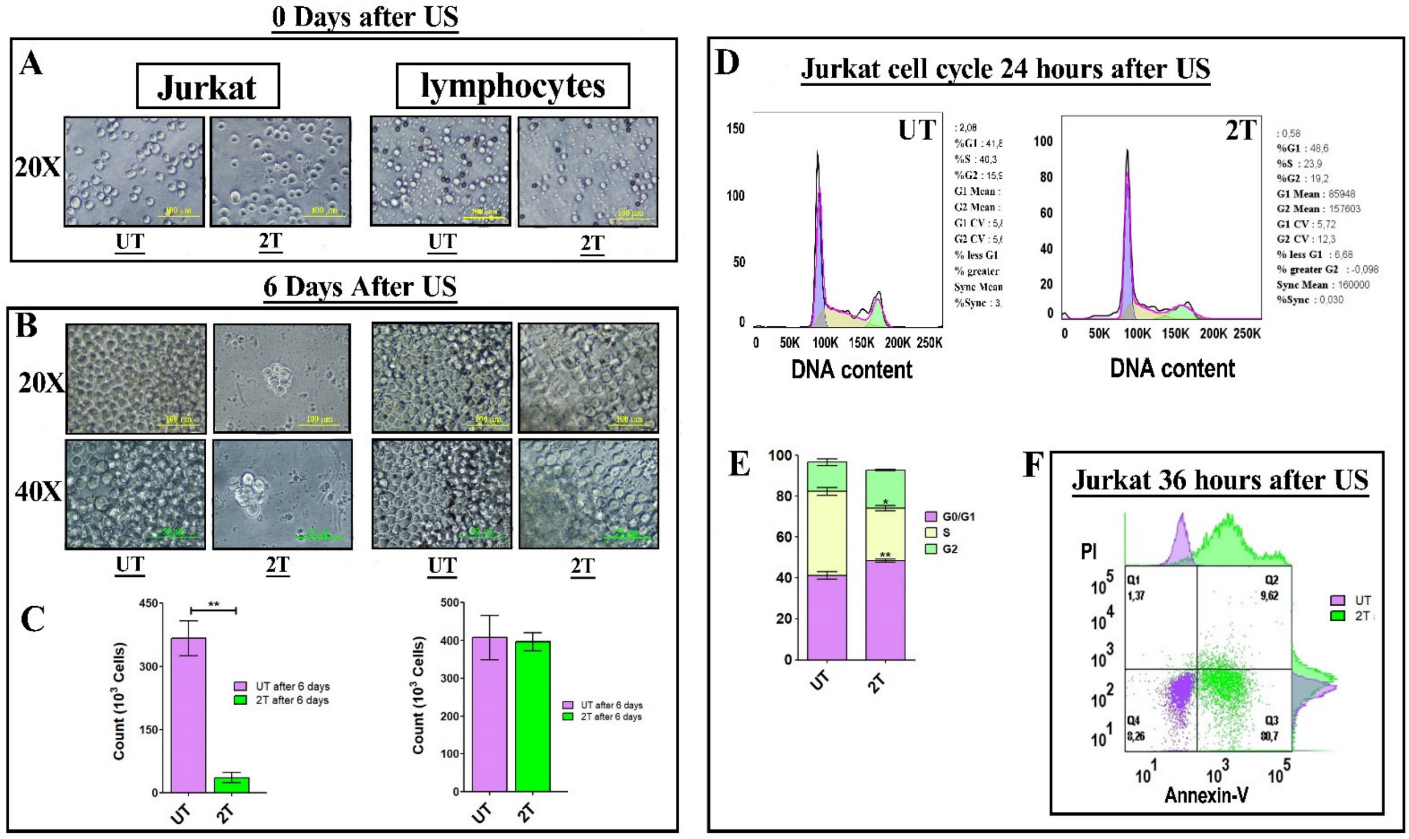
** (A and B) left panels:** 8 x 105 Jurkat cells (UT and 2T) were grown up for 6 days and live cells were counted by trypan blue exclusion. **(C) left panel** histogram of UT and 2T live Jurkat cells after 6 days. UT (purple box) and 2T (green box). **(A and B) right panels** 8 x 105 lymphocytes (UT and 2T) were used as control and grow-up in the presence of CD3/CD28 beads and IL-2, 6 days post-US treatment. **(C) right panel** histogram of UT and 2T live lymphocytes after 6 days. UT (purple box) and 2T (green box). The bars represent 100 μm (20X magnification) and 50 μm (40X magnification) of resolution. All Images are representative of three independent samples. T-test (**p<0.001). Data are mean + SEM of three independent experiments.** (D)** shows DNA content of UT **(left)** and 2T **(right)** Jurkat cells were analyzed after 24 hours post US treatment (representative cell cycle plot) Dean Jett-Fox model was used to analyze cell cycle components. **(E)** shows cell cycle phase histogram. G0/G1 (purple box), S (yellow box) and G2/M (green box). **(F)** representative Jurkat dot plot analysis of Annexin-V/Pi assay, 106 cells of UT (purple dots) and 2T (green dots) were analyzed 36 hours after US-treatment.

To confirm of 2T treatment over all leukemia cells (myeloid and lymphoblastic leukemia cell lines) we also performed a US treatment and proliferation assay on Jurkat cells and healthy lymphocytes. Since OCI-AML-3 and MOLM-13 undergo complete necrosis after 24 hours (2T), we performed the same assay on the lymphocyte's counterpart Jurkat (an Acute lymphoblastic leukemia T cell line). Jurkat cells should behave similar to lymphocytes but differ in their US response from AML cell lines as shown in Figure 2A and Figure 2B. Figure 2A, shows the early effect of US at various time exposure on Jurkat cells. Since after 2T treatment the Jurkat cells remain (about 35%) alive, we investigated whether 2T US could be used to induce apoptosis in Jurkat cells using different exposure times. At 2T treatment, Jurkat cells do not show necrotic effect as AML cell lines (Figure 2A). Jurkat cells show soon (0 hours after US treatment) about 65% of mortality (Figure 2A) but 35% of cells remain still alive (2T), and no alive cells were found after 5T (100 s) US treatment. Hence, we evaluated the ability of 2T Jurkat cells to rescue the growth when US stimulus was removed. Therefore, UT and 2T Jurkat cells were seeded at 80000 cells/0.2 ml in 96 well plates, and cells were grown for up to 6 days (Figure 3A and Figure 3B, left panels).

As a control, healthy lymphocytes were grown, after US treatment, in the same condition and in presence of CD3/CD28 activation beads (which can activate proliferation in resting lymphocytes), and the growth was followed up for 6 days (Figure 3A and Figure 3B, right panels). As shown in Figure 3A and Figure 3B, 2T Jurkat cells are not able to proliferate as UT controls. On the contrary, healthy lymphocytes can grow in media supplemented with CD3/CD28 activation beads (as described in materials and methods). These findings agree with some results of other authors; in fact, Jurkat cells are more resistant to apoptosis. Firestein et al. observe that 50% of Jurkat at 750 kHz survived immediately after treatment and that 50% of cells undergo necrosis 38,39. Our data could suggest that Jurkat cells undergo a cell cycle arrest after US treatment. Indeed, we have analyzed 2T Jurkat cell cycle after 24 hours post-US- treatment, and as shown in Figure 3D, and Figure 3E. Jurkat cells, as expected, undergo a cell cycle arrest in G0/G1 phase showing a decrease of S phase and an increase of G1 phase and SubG1 peak was about 10% in 2T and about 2% in UT cells. Finally, Jurkat cells were analyzed by Annexin-V/Pi assay 36 hours post-US treatment. Figure 3F shows an increase of early apoptotic cells (80.7% 2T with respect to UT) and low late apoptosis (about 9% in 2T with respect to UT cells).

### Annexin-V/Pi assay

**Figure 4:**
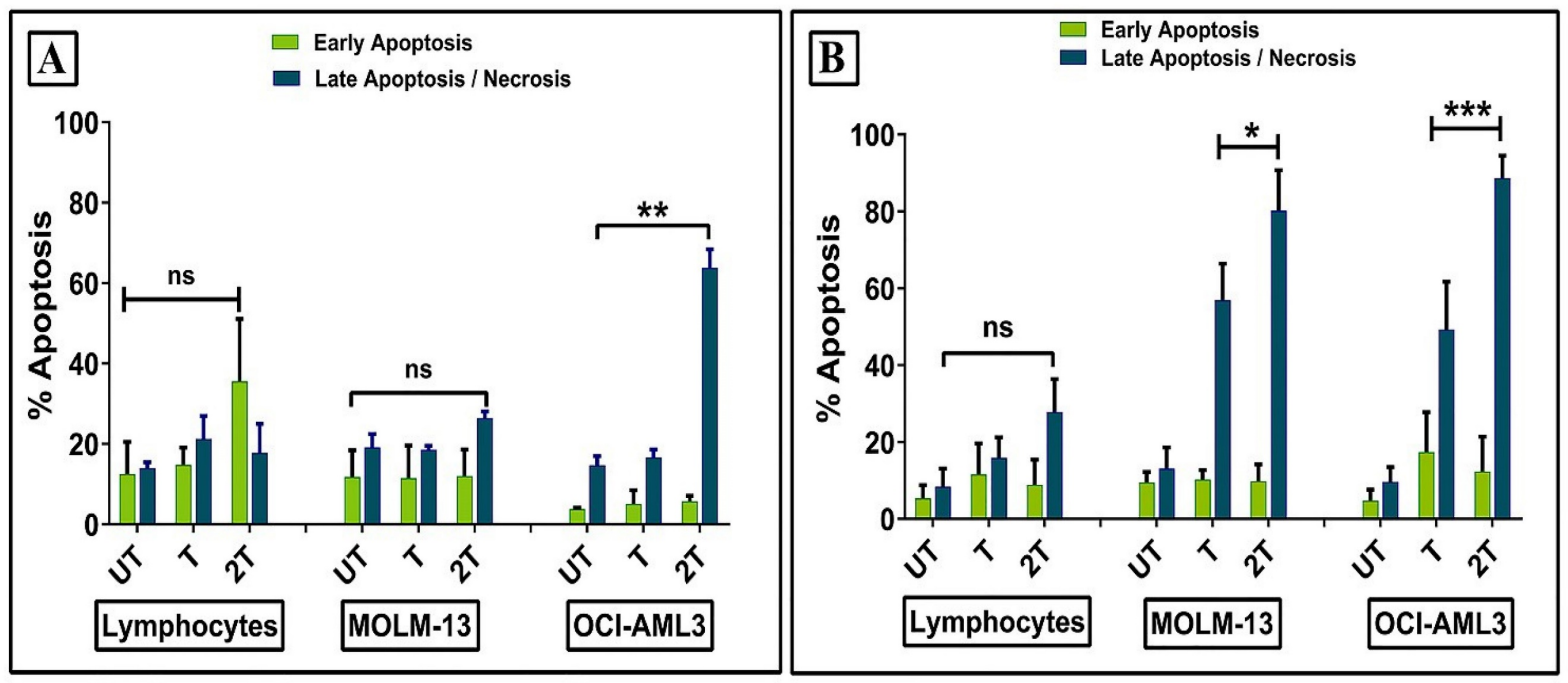
** Effect of ultrasonic treatment on the apoptosis of healthy lymphocytes and leukemia cells (OCI AML-3 and MOLM-13).** Apoptosis was evaluated by Annexin/PI assay after 30 min **(A)**, 24 hours **(B)** posttreatment. UT untreated, T 20 sec 1 MHz, 2T (2x) 20 sec 1 MHz. Results are mean +/- SD of three independent experiments **(A)** and + SD of 5 independent experiments** (B)**. For late apoptosis unpaired t-test with Welch's correction was used for **(A)** (** p< 0.001) and (B) (** p< 0.001), (* p< 0.05).

In order to clarify how ultrasound affects the survival of healthy lymphocytes and leukemic myeloid cell lines, we evaluated early and late apoptosis/necrosis events at 30 min and 24 hours after sonication. Healthy lymphocytes showed no increase in early and late apoptosis/necrosis 30 min post-treatments compared to the control (UT) (Figure 4A). Similar results were observed for MOLM-13 cell line. This can be explained probably by a different regulation of P53 during the first hours. It has been already demonstrated that DNA repair activity is increased early in P53- induced apoptosis and, when apoptotic stimuli were removed, the apoptosis becomes reversible 37. Hence, probably, the high resistance of lymphocytes to early apoptosis could be attributed to plasma membrane resistance, antiapoptotic protein expression, intracellular ROS scavengers, or more complex mechanisms which include all of them. Only OCI-AML-3 cell line showing high levels of late apoptosis/necrosis were found already after 30 min 2T post-treatment. Figure 4B shows early/late apoptosis after 24 hours post-treatment. No increase in early apoptosis levels was found for all cells, while drastically increased late apoptosis/necrosis levels were found for myeloid leukemia cell lines for both treatments respect to the relative UT controls. In contrast, lymphocytes did not statistically show an increase in late apoptosis/necrotic events.

### Effect of US on viability and mitochondrial membrane potential of lymphocytes

**Figure 5:**
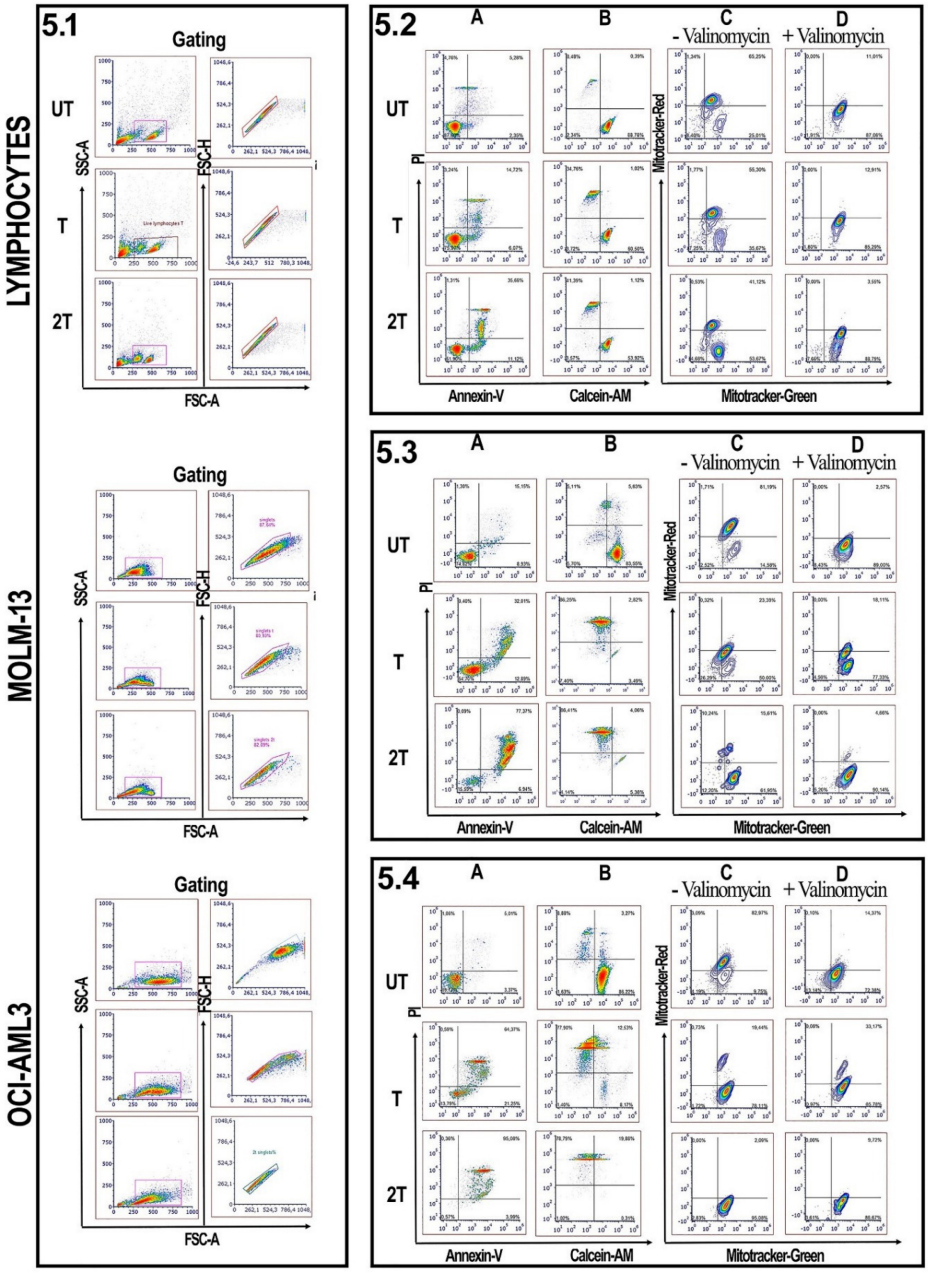
** Effect of ultrasonic treatment on viability and mitochondrial membrane potential: 5.1** Lymphocytes, MOLM-13 and OCI-AML3 were gated on FSC-A/SSC-A. Singlets were gated on FSC-A/FSC-H parameters.** Lymphocytes analysis. 5.2 (A and B)** show lymphocytes Annexin-V/Pi and Calcein/Pi assays. **5.2 (C, D):** lymphocytes were incubated without or with valinomycin 100 nM 10 min 37 °C, and then incubated with Mitotracker Green and Mitotracker Red. Data were represented as contour plots. **MOLM-13 analysis. 5.3 (A and B)** show MOLM-13 Annexin-V/Pi and Calcein/Pi assays. **5.3 (C, D):** MOLM-13 were incubated without or with valinomycin 100 nM 10 min 37 °C, and then incubated with Mitotracker Green and Mitotracker Red. Data were represented as contour plots. OCI AML-3 analysis. **5.4 (A and B)** show OCI AML-3 Annexin-V/Pi and Calcein/Pi assays. **5.4 (C, D):** OCI AML-3 were incubated without or with valinomycin 100 nM 10 min 37 °C, and then incubated with Mitotracker Green and Mitotracker Red. Data were represented as contour plots. UT untreated, T 20 s 1 MHz, 2T (2x) 20 s 1 MHz. All plots are representative of three healthy donor samples. 15000 events were acquired.

Since lymphocytes, as shown in Figure 1 and 4, have a remarkable resistance to US treatments and better control regarding apoptosis events, we evaluated for myeloid leukemia cells and healthy lymphocytes, the effect of US, after 24 hours of treatments, on mitochondrial membrane potential, by measuring the mitochondrial membrane potential and mitochondrial mass as described by Monteiro et al. 30. Figure 5 shows the effect of US on the viability of healthy donor lymphocytes as well as MOL-13 and OCI-AML-3 after (T) or (2T) US exposures. After one treatment (T) no evidence of apoptotic events was observed for lymphocytes (Figure 5.2A). After 2T pulses, very few lymphocytes undergo apoptosis /necrosis. To verify the higher viability of lymphocytes, we incubated, after US treatment, the lymphocytes with a permeable dye (Calcein-AM) and monitored their fluorescence. Calcein AM is a cell-permeant dye that can be used to determine cell viability in most eukaryotic cells. In live cells, the nonfluorescent Calcein AM is converted to a green fluorescent Calcein after acetoxymethyl ester hydrolysis by intracellular esterase. AS shown in Figure 5.2 B, a small decrease in cytosolic Calcein-AM uptake was observed and most Calcein was retained in the viable lymphocytes (89.13% of Untreated (UT) cells, about 58.55% for treated (T) cells, and 54.33% for cells 2T). We have also analyzed the US effects on mitochondria membrane potential. A drop of mitochondrial membrane potential is one of the early steps of the apoptosis events, preceding the DNA fragmentation and phosphatidylserine exposure 40. In order to evaluate how much the US affected mitochondrial membrane potentials, we used two specific mitochondrial dyes, Mitotracker Red for mitochondrial membrane potential and Mitotracker Green for mitochondrial mass. As shown in Figure 5.2 C, one treatment (T) affects very little the lymphocyte's mitochondrial potential, with about 10% loss of functional mitochondria versus control (UT) cells (65% of cells with high mitochondrial potential for UT versus 55% of (T) cells). There was only a slight decrease in mitochondrial potential at 2T about 23% loss of functional mitochondria (42% of cells (2T) with high mitochondrial potential versus 65% high mitochondrial potential of untreated cells (UT)). We used the ionophore Valinomycin, a mitochondrial decoupling molecule, as a positive control (Figure 5.2 D). The results suggest that some lymphocytes maybe could have some fine mechanisms that control apoptotic events in different extents and ways, such as different expression patterns of anti-apoptotic proteins like Bcl-2 family or maybe downregulation of PUMA or NOXA, pro-apoptotic proteins, or P53 17,34,35, or maybe upregulating UCP2 (uncoupling protein 2) 31,41 which in turn lowers the levels of mitochondrial ROS, or maybe in the regulation of ERK1/2 pathways 42. Moreover, some lymphocyte subsets could have high levels of ROS scavenger's molecule such as GSH, etc.

### Effect of US on viability and mitochondrial membrane potential of AML cell lines

On the other hand, leukemia and lymphoblast cells undergo deep changes in their viability, under US treatments 17,33 since they are more susceptible to ROS. Figure 5.3 also shows the US effects on the myeloid MOLM-13 cell line. At (T) treatment, MOLM-13 showed over 50% of late apoptotic/necrotic events and over 70 % for (2T), after 24 hours post-treatment (Figure 4B and Figure 5.3 A). It's interesting to note that MOLM-13 cells show a little resistance to apoptosis; in fact, they show about 47% of cells have high Calcein-AM but at the same time, they have plasma membranes compromised (Figure 1). Furthermore MOLM-13 cell line cannot proliferate after 2 treatments (2T) of US (data not shown). Finally, 2T treatment is more effective in terms of apoptosis/necrosis for myeloid leukemia cells. MOLM-13 has a high loss of functional mitochondrial already at one treatment (T) (Figure 5.3 C). Similar results were obtained with another myeloid cell line OCI-AML-3. Figure 5.4 also shows a marked susceptibility to oxidative stress and apoptosis of the OCI-AML-3 cell line 43. Cells with a large surface area, such as myeloid cells, are more responsive to ultrasound, accordingly with Oncotripsy Theory and Pappalettere 11,6.

### Effect of US on the viability of red blood cells

**Figure 6:**
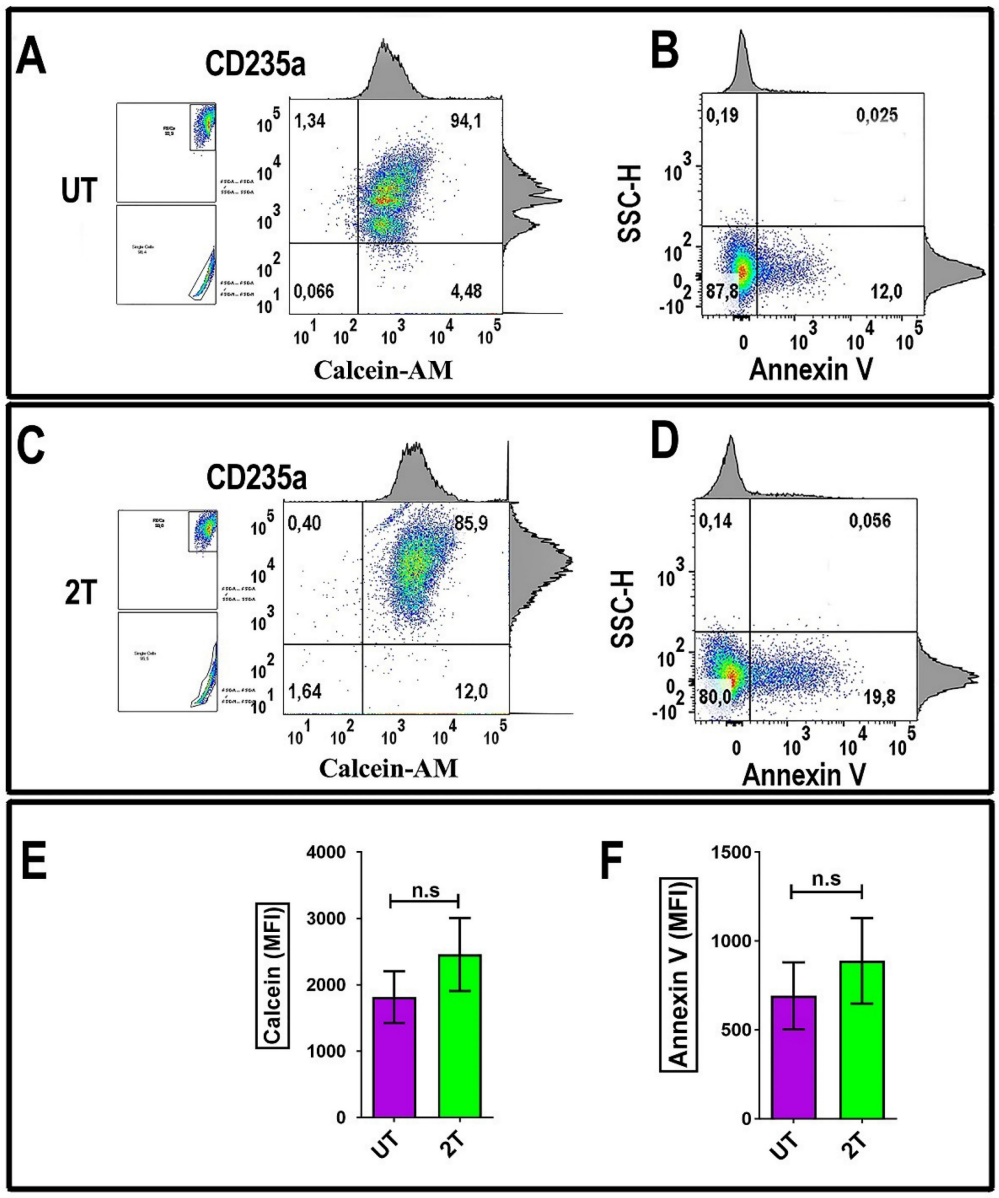
** RBCs Annexin-V and Calcein-AM analysis: (A)** 106 fresh isolated RBCs from were incubated with 5 μM Calcein-AM for 135 min at 37°C. After washing, the cells were incubated with CD235a (glycophorin A) and analyzed by flow cytometry. **(B)** Other Samples (density plots on the right) were incubated after 48 hours from 2T, with Annexin-V and then analyzed by Flow Cytometry. **(C)** 106 fresh isolated RBCs were treated with US and incubated for 24 hours, then when incubated with 5 μM Calcein-AM for 135 min at 37 °C. After washing, the cells were incubated with CD235a (glycophorin A) and analyzed by flow cytometry. **(D)** other aliquots (106 RBCs) were treated with US and incubated after 48 hours with Annexin V and then analyzed by Flow Cytometry. **UT** untreated, **2T** (2x) 20 s 1 MHz. The density plots are representative of three healthy donor samples. 20000 events were acquired. RBCs were gated by log FSC-A/FSC-A and FSC-A/FSC-W for singlets. Ancestry dot plots are indicated on the left. The numbers indicate % of cells. **(E)** and **(F)** relative histograms + SEM of Calcein and Annexin V assay (UT purple, 2T green), respectively relative to RBCs from three healthy donors. All density plots are representative. Unpaired t-test was used for statistical analysis.

To evaluate the effect of US on RBCs, the US-treated cells were incubated with Calcein-AM dye. A method to evaluate the red blood viability (membrane integrity) is measuring Calcein-AM uptake and its retention. As shown in Figure 6C, healthy red blood cells were treated by 2T US and then incubated after 48 hours post-US-treatment with 5 *μ*M of Calcein-AM and then stained with monoclonal CD235a specific antibody for RBCs (Figure 6A and Figure 6C) or with Annexin V (Figure 6B and Figure 6D) to evaluate cell viability. As shown in Figure 6C, RBCs retain most of their Calcein, after two treatments (2T). Finally, alive 2T RBCs didn't show any blebbing or changes in their morphology like schistocytes (Figure 1), red cells could survive to US stress-induced and subsequent sequestration by scavenger cells; indeed, we have seen any increase of Annexin V as shown in Figure 6D of 2T RBCs respect to the control (UT) and Figure 6E, and Figure 6F. Virtually, no significative senescence was observed *in vitro* after 48 hours in 2T samples, since our condition are less severe than indicated by Brayman AA, et al. and Abramowicz SJ, et al. 44,45.

### Expression of CD69, CD137 activation markers in healthy lymphocytes after US treatment

**Figure 7:**
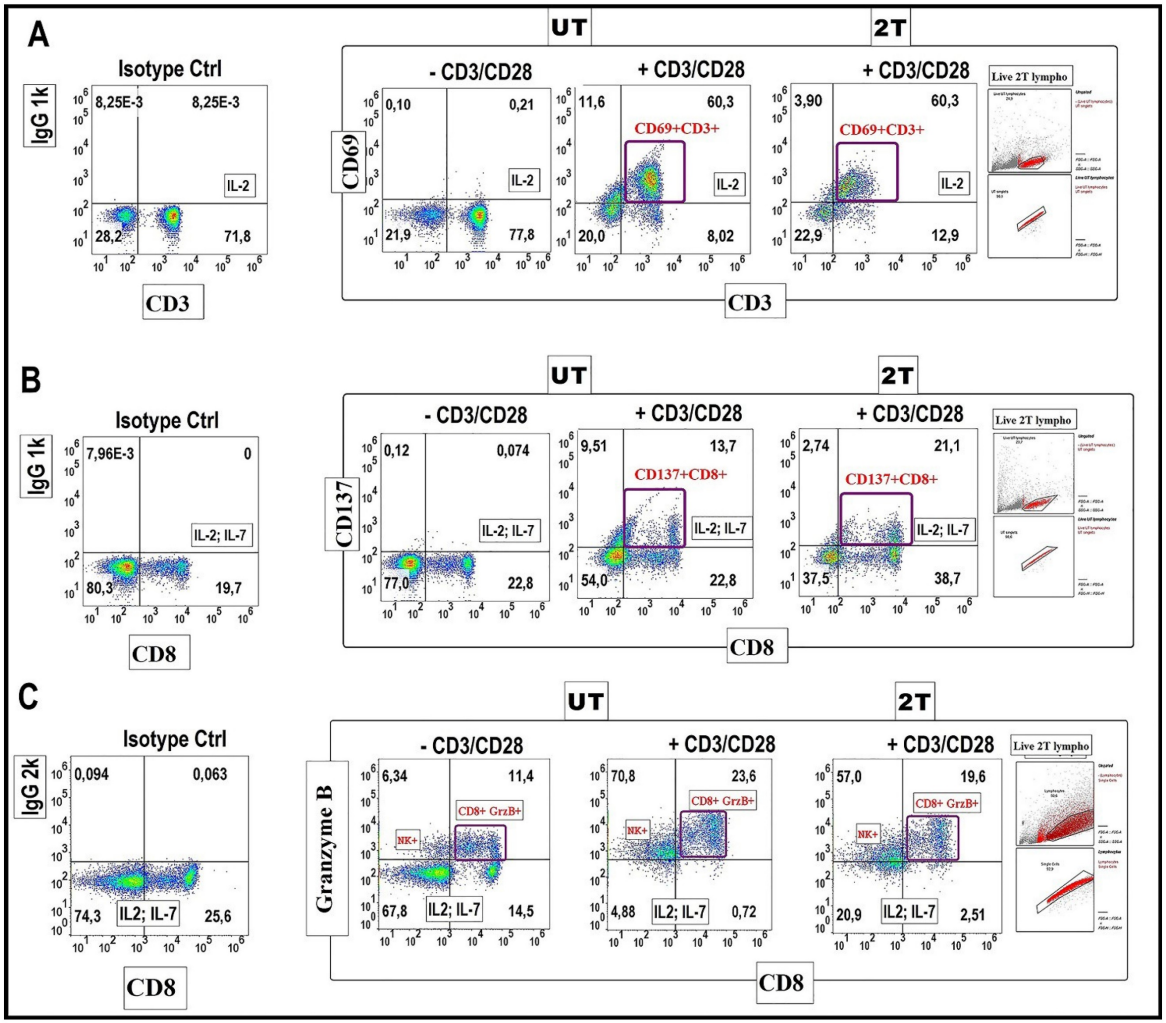
** CD69-CD137 and CD8+ Granzyme B expression analysis**: 106 lymphocytes UT and 2T were incubated after US treatment with 40 U/ml IL-2 and with CD69, CD3 antibodies **(A)** and with 40 U/ml IL-2 and 15 U/ml IL-7, CD137, CD8 antibodies **(B)** in presence or absence of CD3/CD28 Dynabeads, and then analyzed by Flow cytometry. Correspondent isotype controls were used to set correct gating. Live lymphocytes (106 cells) were then identified by FSC-H/SSC-H parameters; singlets were gated by FSC-A/FSC-H. **(C)** For Granzyme B detection 106 lymphocytes, control and 2T, were incubated with 40 U/ml IL-2 and 15 U/ml IL-7 in presence or absence of CD3/CD28 Dynabeads, then lymphocytes were stained for CD8 surface marker followed by Fix and Perm protocol for Granzyme B staining. The density plots are representative of three healthy donor samples. 20000 events were acquired.

Another important aspect is considering the effects of US on the expression of early activation markers such as CD69, or specific CD8+ T cytotoxic effector marker CD137 and Granzymes production. Thus, these markers are essential for the functionality of lymphocytes, and mainly for CD8+ cytotoxic activities. For this purpose, lymphocytes were incubated with antiapoptotic cytokines such as Il-2 or Il-7, as a negative control. These cytokines are important for the survival and proliferation of naïve T and mainly cytotoxic cells 46. Lymphocytes were incubated for 24 hours with IL-2 and in the presence or absence of CD3/CD28 Dyanbeads to stimulate proliferation and activation after (2T) treatment. Figure 7 shows the expression of the early activation marker CD69 (Figure 7A), and NK, CD8+ activation/survival marker CD137 on live lymphocytes (Figure 7B). Isotype controls were used at the same concentration as their respective expression markers. UT and 2T cells, were then incubated for 24 hours with CD3/CD28 Dynabeads and with 40 U/ml IL-2 (Figure 7A) or IL-2 (40U/ml) + IL-7 (15U/ml) (Figure 7B) for *in vitro* stimulation and activation of total CD3 live lymphocytes or CD8 subset, respectively. After removing beads, the cells were incubated with CD69/CD3 (Figure 7A) or CD8/CD137 (Figure 7B) antibodies or their respective Isotype antibodies and then analyzed by flow cytometry. As shown in Figure 7A, live (2T) lymphocytes were still able to produce activation markers CD69 (60.3% CD69+CD3+ vs 60,3% of UT) or CD137 (21.1% CD137+CD8+ vs 13,7 of control UT) and undergone specifically activation by CD3/CD28 stimuli as controls live (UT) lymphocytes. IL-2 and (IL2 + IL-7) alone didn't produce any relevant effect on CD69 and CD137 expression. Thereby, live-resting lymphocytes are still able to produce activation markers indicating that genome functionality is not compromised.

### Expression of Granzyme B in healthy lymphocytes after US treatment

Finally, we tested the production of Granzyme B by CD8+cells after 2T treatment (Figure 7C). Briefly, UT and 2T- treated lymphocytes were incubated with Dynabeads for 48 hours and with protein transport inhibitor (4 hours before the analysis), then fixed and stained for surface marker CD8 and intracellular Granzyme B. Figure 7C show that Granzyme B granules formation, after beads stimulation, is similar in both UT and 2T lymphocytes; this indicates that lymphocytes not only are able to proliferate after US treatment but also have a good polarization and finally CD8+ subset cells with normal cytotoxic activity. Furthermore, a remarkable NK Granzyme B expression (upper left quadrants on density plot stimulated cells by CD3/CD28) is also visible after ultrasound treatment demonstrating indirectly that the innate immune cells system isn't compromised at all by US treatments.

### Proliferation of healthy lymphocytes after US treatment

**Figure 8:**
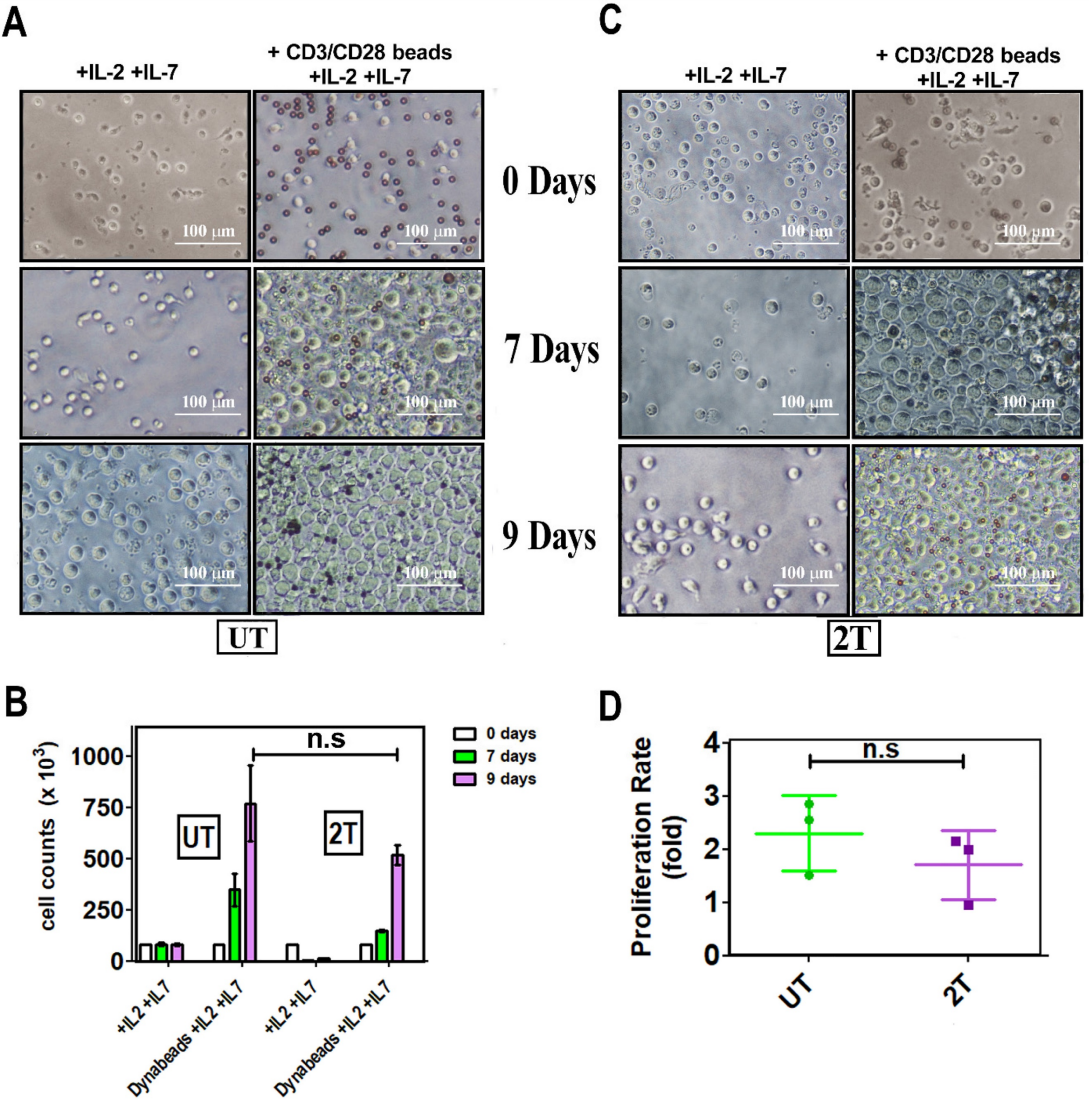
***In vitro* expansion of T cell upon beads-induced activation: A (UT) and C (2T) representative images**. PBMC aliquots from at least 3 healthy donors were used for US treatment. UT and 2T cells (8 x 105 cells/ well), were incubated with 40 U/ml IL-2 and 15 U/ml IL-7 in the presence or absence of CD3/CD28 Dynabeads. Responder cells of all experimental conditions were counted at indicated days after stimulation and after US treatments. The bars represent 100 μm at 20X magnification. **(B)** The histogram shows live lymphocytes expansion after US treatment after 7 days (green box) and 9 days (purple box). **(D)** Proliferation ratio is described in material and methods. UT (green) and 2T(purple). n.s not significative. Data are mean + SEM.

To assess the ability of lymphocytes to proliferate after US treatments, the cells were incubated, with CD28/CD3 beads, and the growth has been followed for 9 days. Figure 8A, B, and C, show that live UT and 2T lymphocytes are effectively stimulated by Dynabeads not only by expressing activation markers but they can actively proliferate. Il-2 and Il-7, alone, activate for proliferation neither UT nor 2T cells; Only Beads stimulated cells were able to expand themself more efficiently, and UT and 2T cells show no differences in proliferation ratio (Figure 8D). IL-7 is mainly critical for homeostatic proliferation and survival of naïve T cells, which can be activated by TCR-CD3/CD28 stimuli more efficiently and then undergo polarization in terms of effector and memory T cells 46, 47.

### ROS production in healthy lymphocytes and Jurkat cells after US treatment

ROS production during cavitation induced by the US treatment is one important factor to be considered. Apoptosis events in cancer cells, after US treatments, have been demonstrated by many authors 14, and the increase of intracellular ROS has been seen for tumoral cell lines 33,43, the which finally determines cell death. It has also been demonstrated that ROS production is important for lymphocytes development and differentiation, under certain conditions 47. Furthermore, T-cell resistance to ROS is quite different, depending on the subset of lymphocytes following this order Teff > Treg > Tnaïve > Tmem 48.

**Figure 9:**
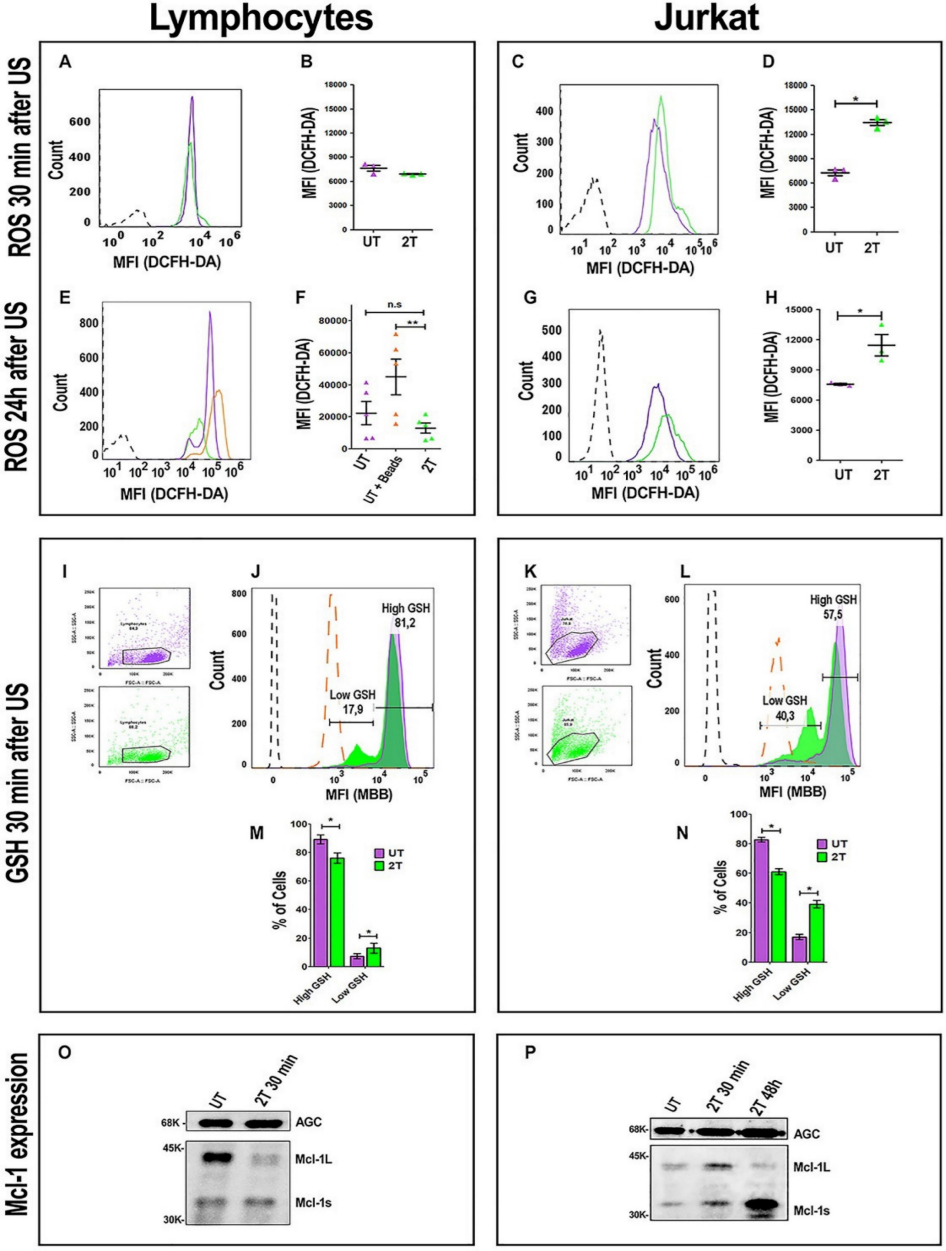
** ROS, GSH production and Mcl-1 expression after US treatment.** ROS levels were analyzed after 30 min. **(A)** Representative histogram plot of ROS content 30 min post-US, UT (purple line) 2T (green line). **(B)** Histogram of lymphocytes ROS 30 min post-US. **(C)** Representative Jurkat histogram plot of ROS content 30 min post-US, UT (purple line) 2T (green line). **(D)** Histogram of Jurkat ROS 30 min post US. **(E)** Representative histogram plot of lymphocytes ROS content after 24 hours post-US. UT (purple line), UT+ beads (orange line) and 2T (green line). **(F)** Histogram of lymphocytes ROS 24 hours post-US. UT (purple triangles), UT+ beads (orange triangles) and 2T (green triangles) **(G)** Representative plot of Jurkat ROS 24 hours post-US. UT (purple line) and 2T (green line). **(H)** Histogram of Jurkat ROS 24 hours post-US. UT (purple triangles), 2T (green triangles). **(I)** Lymphocytes FSC-A/SSC-A dot plot gating strategy, UT (purple dots) 2T (green dots). **(J)** Representative lymphocytes histogram of GSH content 30 min post-US. UT (purple line), 2T (green line), UT + 100 μM NEM (orange large- dashed line), unstained (black dashed line). **(K)** Jurkat FSC-A/SSC-A dot plot gating strategy, UT (purple dots) 2T (green dots). **(L)** Representative Jurkat histogram plot of GSH content 30 min post-US. UT (purple line), 2T (green line), UT+ 100 μM NEM (orange large-dashed line), unstained (black dashed line). **(M)** Histogram of lymphocytes GSH content 30 min post-US. UT (purple box), 2T (green box). **(N)** Histogram of Jurkat GSH content 30 min post-US. UT (purple box), 2T (green box). ROS were measured on live non apoptotic cells. Data are mean + SEM of three independent experiments. Unpaired T-test was used (*p<0.05) and (**p<0.001). **(O)** Western blot of lymphocytes Mcl-1 expression 30 min post-US treatment. AGC (Aspartate/Glutamate carrier) for protein normalization. **(P)** Western blot of Jurkat of Mcl-1 expression 30 min and 48 hours post-US treatment. AGC (Aspartate/Glutamate carrier) for protein normalization.

To evaluate if a possible increase in ROS production by US treatments could affect lymphocyte viability, we set up the experiment using two controls. Figure 9A shows the ROS levels in healthy lymphocytes after 30 min and 24 hours post 2T US-treatment. After 30 min (Figure 9A and Figure 9B), total ROS levels of 2T-treated lymphocytes were identical to the control. Furthermore, we have evaluated maximal physiological ROS production and their tolerance. For this purpose, we used the Dynabeads for cell activation and activated lymphocytes as a positive control, since activation of lymphocytes by CD3/CD28 beads or by macrophages/dendritic cells shows a physiological increase of ROS 48-50 without affecting proliferation, while untreated cells were used as basal ROS control (Figure 9E and Figure 9F). The untreated lymphocytes (basal ROS) and UT lymphocytes incubated with Dynabeads UT (UT+ Dynabeads) were grown-up for 24 hours and then analyzed by measuring DCFH-DA fluorescence. As shown in Figure 9E and Figure 9F, after 2T treatments, lymphocytes do not show any increase in total ROS, on the contrary, they have a low level of ROS like the control. Finally, as expected, UT lymphocytes incubated with Dynabeads show a physiological increase of ROS with respect to the control (UT). On the contrary, Jurkat cell increases their ROS levels by about 200% already after 30 min (Figure 9C and Figure 9D), and ROS levels remain high also after 24 hours, with respect to the UT control (Figure 9G and Figure 9H).

### GSH content in healthy lymphocytes and Jurkat cells after US-Treatment

It would be conceivable that during the first hours, lymphocytes can up-regulate the expression of their ROS scavenger genes more efficiently than cancer cells, since they have better control of ROS production. To evaluate this hypothesis, we also analyzed GSH content after 30 min post-US treatment. Figure 9I and Figure 9K, represent gating strategy for lymphocytes and Jurkat cells, respectively. After 2T US-treatment, lymphocytes have about 81.2% of cells (with respect to untreated control) with high GSH content, and only 17.9% of cells with low GSH content as shown in Figure 9J and Figure 9M. On the contrary, Jurkat cells, in the same experimental conditions, have only 57.5% of cells with high GSH content and about 40.3% of cells with low GSH content (Figure 9L and Figure 9N). Clearly, a loss of about 30% of GSH and high ROS levels could heavily unbalance the redox state of Jurkat cells, indeed this could determine 24 hours a cell cycle arrest in G1 (Figure 3D and Figure 3E).

### Mcl-1 expression in healthy lymphocytes and Jurkat cells after US treatment

Since survival of immune cells depends on anti-apoptotic Bcl-2 family members, we have evaluated the involvement of anti-apoptotic Mcl-1 protein. Mcl-1 has a very short half-life, ca. 30-40 min 51. Furthermore Mcl-1 has two active isoforms: Mcl-1_L_ (anti-apoptotic) and Mcl-1s (pro-apoptotic). Hence, we have evaluated the expression of Mcl-1 after 30 min post-US treatment by western bot analysis. Figure 8O and Figure S1, show a loss of lymphocytes Mcl-1_L_ (anti-apoptotic) expression after 2T treatment, but no increase of Mcl-1s (pro- apoptotic) isoform was detectable. Probably, Mcl-1_L_, under ultrasound stress, undergoes degradation or phosphorylation/ubiquitylation followed by proteasome degradation in healthy resting lymphocytes. Maybe there are other mechanisms that prevent late apoptosis in lymphocytes (i.e., Bcl-2, Bcl-xl overexpression, etc.). Our findings show that Mcl-1 is not involved in the first step of lymphocyte survival after US treatment since Mcl-1_L_ are undetectable but no increase of Mcl-1s was found. On the contrary, Jurkat cells have a little increase (about 1.5-fold) of Mcl-1_L_ during first 30 min after US-treatment respect to UT cells, and an increase (about 1.7-fold) of Mcl-1s (Figure 9P and Figure S1). Finally, after 48 hours post US-treatment, Jurkat cells undergo a decrease of Mcl-1_L_ and increase of Mcl-1s isoform (about 5-fold) (Figure 9P).

## Discussion

In this work we demonstrate that PBMC, CD3/CD8 and NK (indirectly) lymphocytes viability and activity is unaffected by ultrasound treatment in agreement with Lagneaux L, et al 15 and Saliev T, et al. 16. On the other hand, we demonstrated, for the first time, that ultrasound not only does not affect healthy CD3/CD8 lymphocyte viability, but their functions and activation are fully preserved, suggesting the absence of any genotoxic effect after US-treatment; furthermore, we demonstrated that in our experimental condition, different leukemic cells, which differ in their lineage and genotypes (AML or ALL cells), could be affected differently by US treatment.

Since T and NK cells can proliferate and differentiate into effectors cells, after US treatment (Figures 7, and 8), we are confident that neither naïve nor differentiated cytotoxic CD8+ cells are affected by US. In light of these data, lymphocytes could activate other contrasting mechanisms to keep low ROS overproduction generated by cavitation, by increasing other scavenger enzymes. Alternatively, their survival could be accounted for the up-regulation of anti-apoptotic Bcl-2 family members such as A1, Bcl-XL, upregulation of ERK1/2, and FAK cascade, etc. 52. It would be interesting to clarify what other mechanisms could occur for the survival of healthy lymphocytes upon US-treatment. As expected, healthy lymphocytes keep the ROS at a basal/physiological level after ultrasound treatment already after 30 min. ROS overproduction in the first minutes could probably activate the Nrf2-mediated response to oxidative stress more efficiently than myeloid cancer cells, upregulating the transcription of numerous ROS-detoxifying enzymes such as glutathione peroxidase 2 (Gpx2) and HO-1 (Heme Oxidase), etc. 53. It has been demonstrated that low energy ultrasound can stimulate Piezo1 protein on lymphocytes with a subsequent increase of cytosolic Ca^2+^. This could activate the Nfat transcription factor which in turn raises the IL-2 level by interacting with its promoter 54, as an autocrine-antiapoptotic function.

Besides, it is known that cancer cell lines have an impaired detoxifying system and are also more susceptible to high ROS levels 55,56. In fact, in AML cell lines, high ROS can downregulate SOD and GSH content as well as Bcl- 2, while pro-apoptotic BAX protein is upregulated 55,56. Differently, lymphocytes could integrate stress signals response using different pathways. Mild to moderate ROS activate calcineurin/NFat and/or Nrf2 pathways bringing to the antioxidants/antiapoptotic system activation, this leads to an increase in Bcl-2, Il-2, GSH, SOD, and HO-1.

Moreover, cancer cells are more susceptible to microtubules and other cytoskeleton components damage caused by ultrasound without affecting healthy cells 57. Another important aspect is the role of p38 and its phosphorylation in the p38 MAPK pathway during US treatment. It has been demonstrated that MOLM-13 after high ultrasound treatment, activates p38, p-CREB/ATF-1, and p-ERK1/2 phosphorylation during the first 5 min after US treatment, and their phosphorylation depends directly on the US intensity used (from mild-moderate, to high) and this effect is more accentuated when MOLM-13 cells were incubated with Sonazoid (lipid-coated microbubbles); on the contrary, p38, p-ERK1/2 expression-phosphorylation in lymphocytes are not detectable, suggesting that ultrasound signaling pathways for healthy lymphocytes are different from cancer cells showing more resistance to US treatment 52. This suggests that, in healthy lymphocytes, ROS content doesn't reach high levels to promote p38 full activation and apoptosis.

Furthermore, AML and ALL cells have a lower buffering capacity against ROS (endogenous and generated by US cavitation) showing an inadequate antioxidant status. This results in an imbalance in the redox microenvironment leading to high oxidative stress (OS) and making them more sensitive with respect to healthy lymphocytes 58. For AML and some ALL cells, most ROS production could be counted (about 60%) by NOX (NADPH oxidase) activity 58 and, since some cancer cells have an impaired antioxidant system, the ROS overproduced by ultrasound has a more negative effect on viability leading to unbalance oxidative status and high oxidative stress causing caspases activation, mitochondrial damage, and finally late apoptosis.

In addition, Jurkat cells in our conditions, have constant high ROS overproduction, low GSH levels, and undergo a G0/G1 phase cell cycle arrest and stop growing. In contrast, US on myeloid cancer cells have a more pronounced and drastic effect, indeed they undergo complete necrosis. Differently, healthy lymphocytes keep, after US-treatment, basal ROS level, have very low depletion of GSH content and can actively proliferate and differentiate after stimulation. Moreover, it has been already demonstrated that Jurkat cells have low levels of SOD2 with respect to healthy lymphocytes, making the cells more sensitive to ROS production 59. Probably other compensative mechanisms are triggered after the initial steps. Recent studies have pointed the attention to the bio- effect of US on granulocytes and monocytes, as shown by Alfred C.H. YU, et al. 60, who found a significant loss of viability mainly in granulocytes, after microbubble-US treatments, but their conditions are different from ours.

## Conclusion

**Figure 10:**
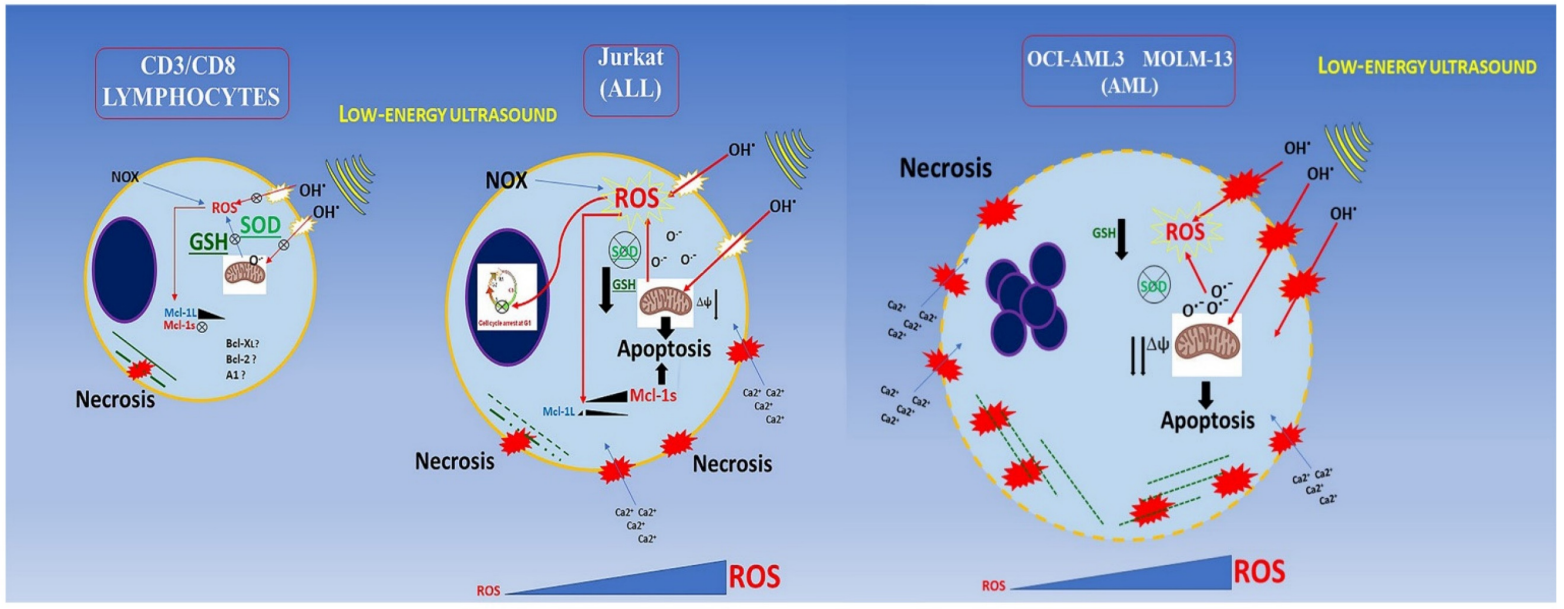
Conclusion schema of this paper. AML and ALL cells have a lower buffering capacity against ROS (endogenous and generated by US cavitation) showing an inadequate antioxidant status.

Low energy ultrasound has different effect depending on cellular type in agreement with oncotripsy theory. Myeloid cells are more severely affected by US treatment; they exhibit necrotic effects. As a result of ROS overproduction, impaired detoxification, and loss of GSH in agreement with results obtained by Lagneaux et al., for the myeloid leukemic cell lines and primary leukemic cells obtained from patients 15 as shown in Figure 10. We extended our analysis to the lymphoblastic Jurkat cells (Acute lymphoblastic leukemic T-cells) which are less susceptible to necrosis but show a G0/G1 arrest following US treatment. After US treatments, CD3/CD8+ lymphocytes are able to proliferate and differentiate into effector cells when properly stimulated *in vitro*. Furthermore, we demonstrated that the expression of MCL-1s (pro-apoptotic isoform) is increased gradually during first 30 min and by five times after 48 hours post-US treatment in Jurkat cells, which in turn increases drastically apoptosis. By contrast, PBMC does not rise either MCL-1L (anti-apoptotic isoform) or MCL-1s; therefore MCL-1 is not involved in lymphocytes survival during US-treatments. In addition, no increase in senescence or apoptosis events is observed in RBC following US-treatment.

It would be interesting to test whether autologous plasma or sera can improve the efficacy of US treatments towards AML or ALL cells (directly isolated from patients) since human serum could confer different viscosity property respect to culture media used for the *in vitro* experiments. Further analysis will be conducted, such as co-culturing with AML cells after US treatments, to determine NK or CD8+ lymphocyte cytotoxicity. However, deep analysis needed regarding the viability and functionality of other lymphocyte subsets like CD4, CD19, Tregs, or Neutrophile granulocytes. Finally, further study would be required to identify what other anti-apoptotic proteins and detoxifying enzymes might also contribute to lymphocyte survival after US -- treatment.

## Supplementary Material

Supplementary figure s1.

## Abbreviations

US: Low-energy ultrasound
MMB: MonoBromobimane
ALL: acute lymphoblastic leukemia
AML: acute myeloid leukemia
RBCs: red blood cells
ROS: reactive oxygen species
PBMC: Peripheral Blood Mononuclear cells
