# Supplementary material for: In Vitro Effects of Low-energy Ultrasound Treatment on Healthy CD3/CD8+ Lymphocytes, Red blood cells, Acute Myeloid leukemia cells, and Jurkat cell line
Source: J Cancer. 2023 Apr 24;14(7):1088–106. doi: 10.7150/jca.83050 (PMC10197932; doi:10.7150/jca.83050)
Supplement: Supplementary file 1 — Supplementary figure s1. [file jcav14p1088s1.pdf]

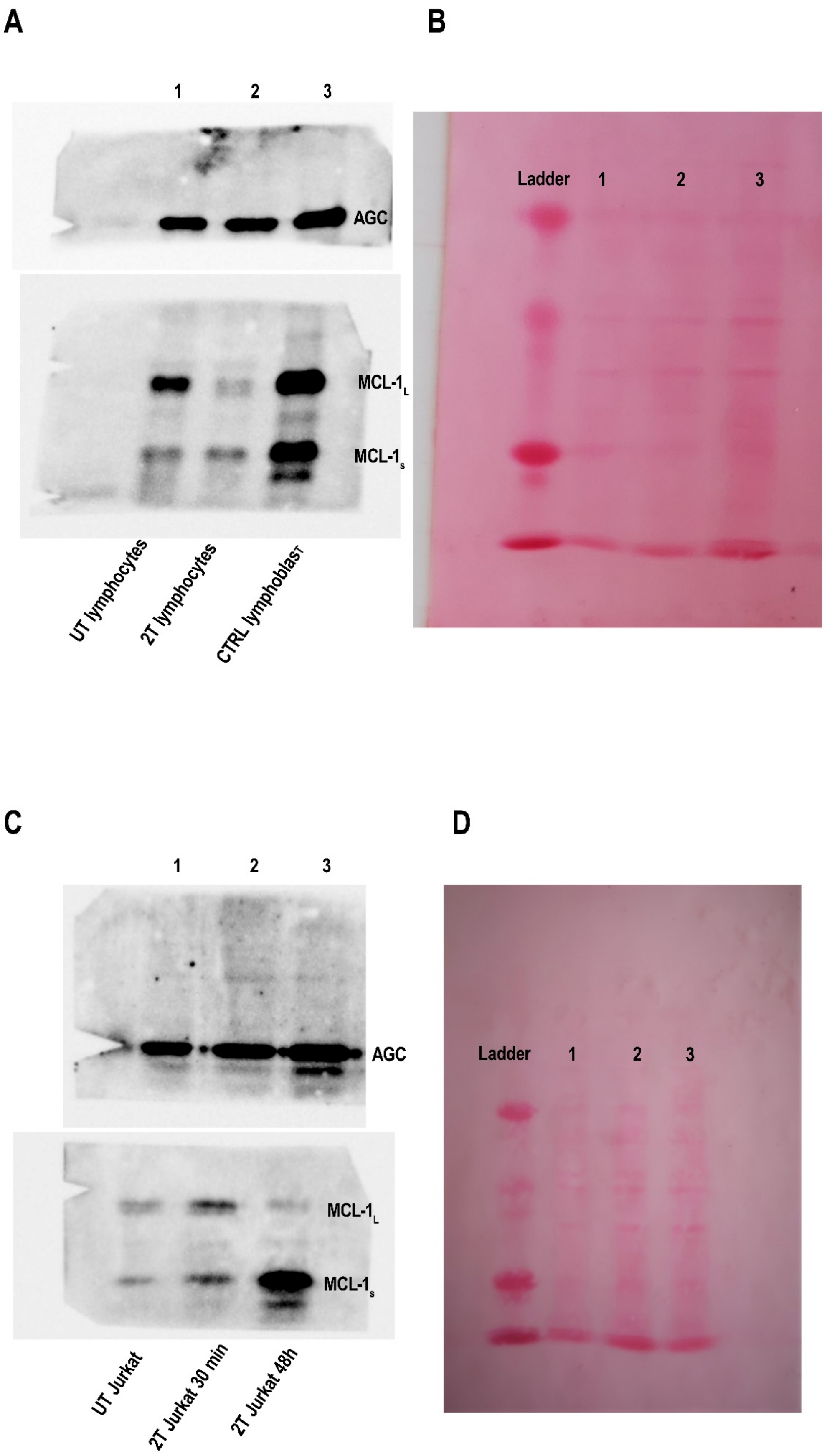

Supplementary figure 1. A Western Blot of Mcl-1 and AGC (housekeeping) of Lymphocytes UT (lane 1), lymphocytes 2T 30 min after US treatment (lane 2). As quality check of MCL-1 antibody we used untreated lymphoblast cell line (line 3). **B** relative red ponceau. **C** Western Blot of Mcl-1 and AGC (housekeeping) of Jurkat UT (lane 1), Jurkat 2T 30 min after US treatment (lane 2), and Jurkat 48 hours after Us treatment (line 3). **D** relative red ponceau.
